# Headache improvement after intracranial endovascular procedures in Chinese patients with unruptured intracranial aneurysm: A prospective observational study

**DOI:** 10.1097/MD.0000000000006084

**Published:** 2017-02-10

**Authors:** Linjing Zhang, Yunxia Wang, Qingkui Zhang, Wei Ge, Xiancong Wu, Hai Di, Jun Wang, Xiangyu Cao, Baomin Li, Ruozhuo Liu, Shengyuan Yu

**Affiliations:** a Department of Neurology, Chinese PLA General Hospital, Beijing, PR China; b Department of Neurology, the First Hospital Affiliated to the Chinese PLA General Hospital, Beijing, PR China; c Department of Neurology, Hainan Branch of Chinese PLA General Hospital, Sanya, PR China.

**Keywords:** headache, intracranial endovascular procedure, unruptured intracranial aneurysms

## Abstract

The aim of this study was to investigate whether there is a long-term improvement in headache of patients with unruptured intracranial aneurysms (UIAs) treated with intracranial endovascular procedures.

Using a prospective design, consecutive patients with UIAs with neuroendovascular treatment from January 2014 to December 2014 were asked to participate. Headache outcomes were established before aneurysm treatment and for 6 months following treatment. Factors associated with different headache outcomes were investigated.

Ultimately, 58 patients completed the 6-month follow-up. In total, 29 patients had preoperative headache. Six months after the intracranial endovascular procedure, 13 patients (44.8%) stated that their headaches were relieved after endovascular treatment; headache in 1 patient improved slightly, and 12 reported disappearance of headache and marked improvement. Overall, the mean headache scores of 29 patients improved on the self-reported Numeric Rating Scale (NRS) after endovascular treatment (6.00 vs. 2.30; *P* < 0.001). Patients with pretreatment tension-type headache, more severe headaches, stent-assisted coiling, and stent implantation of the aneurysm were the important disadvantage for patients in improvement of post-procedure headache.

Treatment of UIAs resulted in relief of headaches in about half of patients who had headaches pre-operatively.

## 1 Introduction

Unruptured intracranial aneurysms (UIAs) have a prevalence of 3% to 6% in the >30-year-old-population.^[1]^ Many studies have reported that chronic headache is the most or one of the most common presenting symptoms in 18% to 36% of cases in larger population studies.^[2–5]^ Thus, patients with UIAs expect improvement of headaches after endovascular treatment for a UIA. Studies show a wide variability in headache responses following intracranial aneurysm treatment. No consensus exists regarding an association between headache and aneurysm. Several studies on headache outcomes in patients with UIAs who underwent endovascular treatment suggest that the majority of patients have improved severity or frequency after treatment. However, a similar number of studies have reported worsening and development of new-onset headache after embolization.^[6–11]^ Additionally, most of these studies were based on small patient populations, and pre- and post-treatment headaches were diagnosed according to the criteria of the second edition of the International Classification of Headache Disorders (ICHD-II), rather than the ICHD-III beta criteria. Here, the aim of this study was to detect whether there is a long-term improvement in chronic headache of patients with UIAs treated with intracranial endovascular procedures.

## 2 Materials and methods

This project was approved by the Research Ethics Committee of the Chinese People's Liberation Army (PLA) General Hospital. All patients provided written informed consent.

This prospective study design has been described previously.^[12]^ In brief, adult patients with planned treatments for UIAs at our Hospital were asked about participating in this study conducted between 2014 and 2015. We used a semistructured questionnaire including 19 questions soliciting detailed information about changes in headache intensity, quality, location, and duration with predefined time intervals: preprocedural headache, 24 hours, 72 hours, 1 week, 2 weeks, 1 month, 3 months, 6 months.

**Figure 1:**
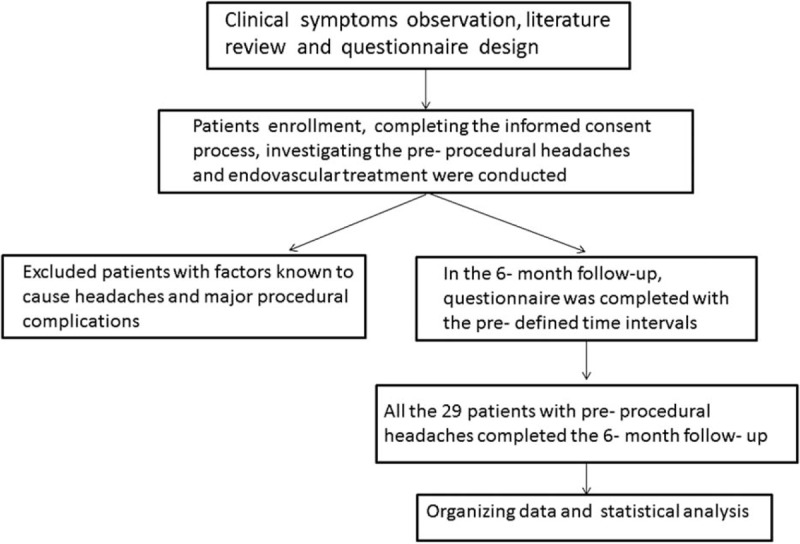
Study design.

When the 6-month follow-up completed, we focused on the status of headache improvement at the time point of 1 month, 3 months, and particularly, 6 months. For the patient ambiguous answer, we record the higher score. For example, if one patient say “either 4 or 5,” we record 5, and factors associated with different headache outcomes at 6 months were investigated. In this study, “markedly improved headaches” were defined as scores that increased by 3 to 5 points (numeric rating scale, NRS). The study design was presented in Figure 1.

### 2.1 Statistical analysis

Descriptive statistics were used to describe patient demographics, headache histories, and aneurysm characteristics. The Wilcoxon 2-sample test was performed to determine changes in mean headache severity pre- and post-aneurysm endovascular treatment. Characteristics of patients with headache improvement following treatment were compared to those with no improvement following treatment using unpaired 2-tailed tests, independent-sample Mann–Whitney *U* tests, or Pearson *χ*^2^ tests as appropriate. *P* values <0.05 were considered significant. Statistical analyses were performed using SPSS ver. 17.0 (SPSS Inc, Chicago, IL).

## 3 Results

In total, 73 patients were enrolled and 8 patients were excluded at the beginning of follow-up because of an arteriovenous malformation (n = 1), an aneurysmal subarachnoid hemorrhage (n = 3), severe head injury (n = 2), and refusal to be followed or inability to describe the headache exactly (n = 2). Among 65 patients, 7 patients were lost to follow- up. Ultimately, 58 patients completed the trial, and 29 patients were preprocedural headaches (NRS scores of 3–10). The female was 12 (41.4%) and male was 17 (58.6%). The mean age was 51.17 (range from 32 to 74) years. Symptoms leading to aneurysms diagnosed were listed as following: recurrent headache, 19 (65.5%); vertigo or vomit, 1 (3.4%); syncope, 3 (10.3%); cerebral infarction, 3 (10.3%); unexpectedly found in a check-up, 2 (6.9%); others, 3 (10.3%). Modalities of treatment were coiling embolization, 7 (24.1%); stent-assisted embolization, 19 (65.5%); stent implantation, 3 (10.3%). The detailed clinical and angiographic characteristics were presented in Table 1.

**Table 1:**
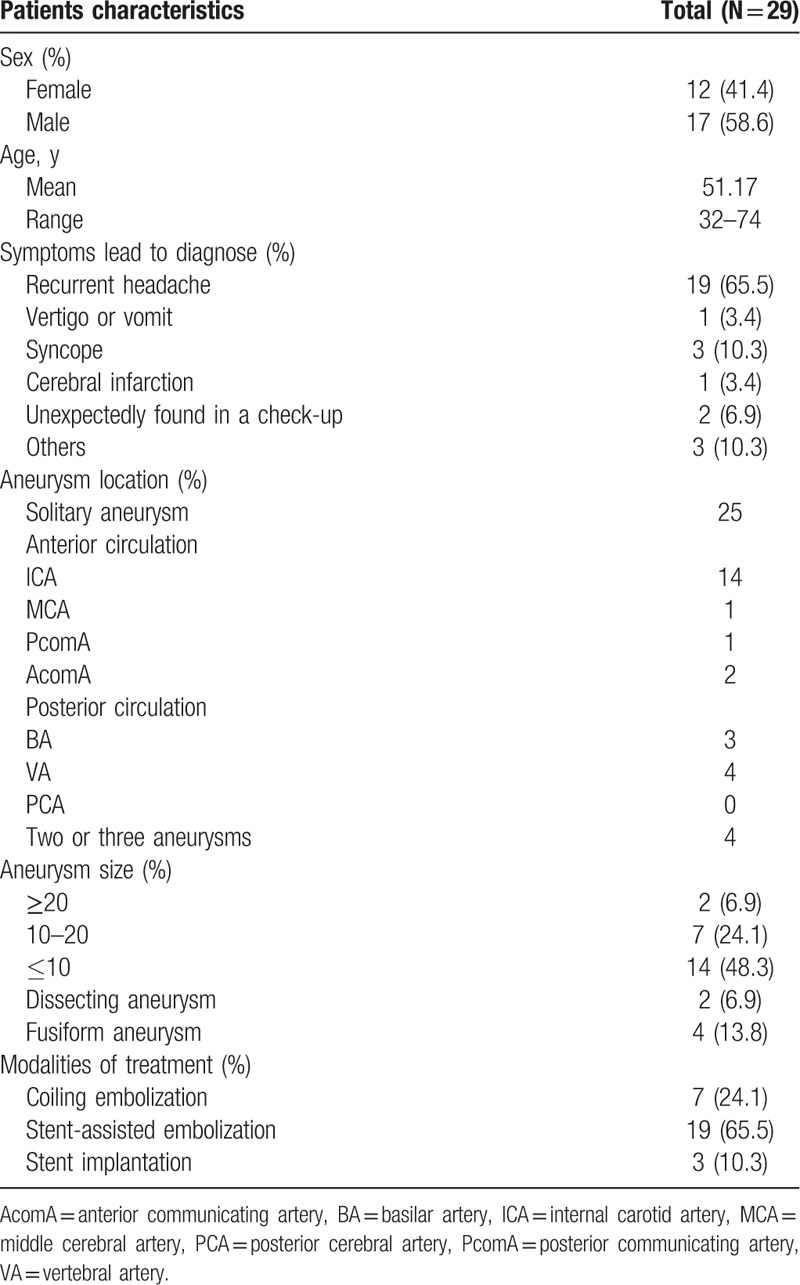
Clinical and angiographic characteristics of 29 patients.

At enrollment, the mean NRS of the 29 preprocedural headaches was 6.00. In Table 2, the characteristics of the 29 preprocedural headaches including frequency, duration, location, quality, intensity, and whether influenced by activity were listed. Thirteen patients (44.8%) experienced headache daily, 14 (48.3%) patients underwent headache 5 to 15 days per month, and 2 (6.9%) patients had headache less frequent with 15 to 30 days per year. Approximately, half 15 (51.7%) headache intensity were moderate (NRS 4–7), and 9 (31.0%) patients suffered the most severe headache.

**Table 2:**
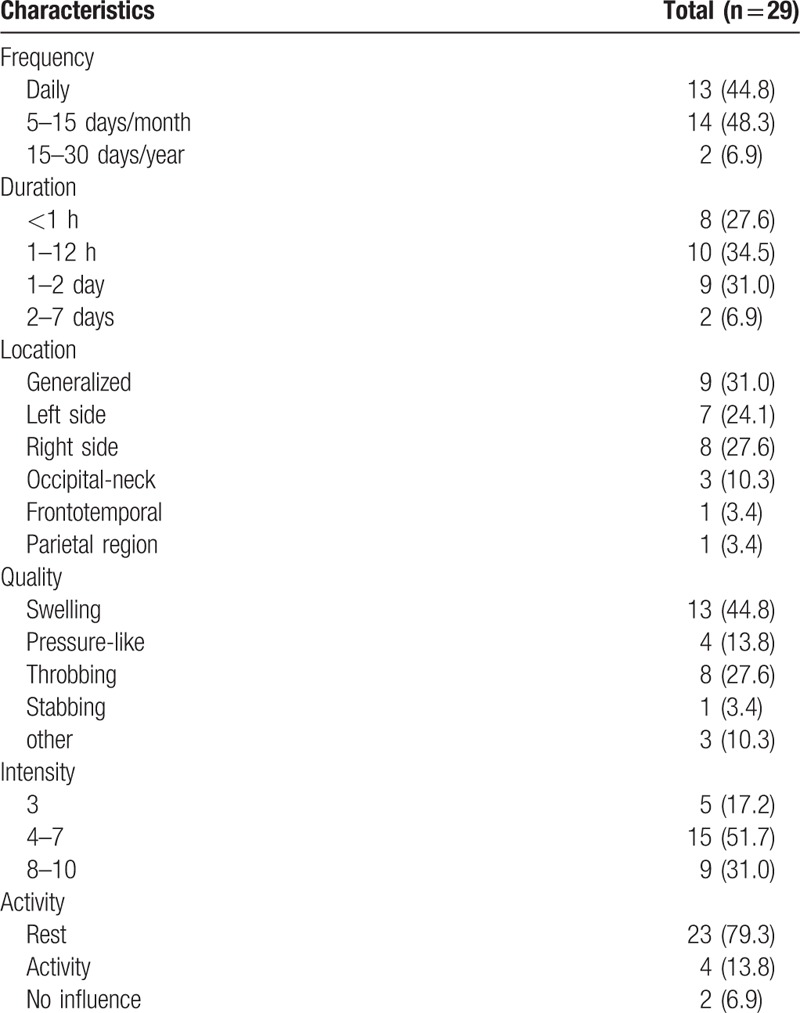
Characteristics of the 29 preprocedural headaches.

There was a continued decline in the number of patients presenting with headache and intensity of headaches within the 6 months’ follow-up (Table 3). One month after the procedure, 9 patients reported that their headache was improved, among which 5 patients’ headache disappeared and 4 patients’ headache markedly improved. The mean headache score was 6.00 preoperatively versus 4.72 at 1 month postoperatively (*P* = 0.7) (Table 3).

**Table 3:**
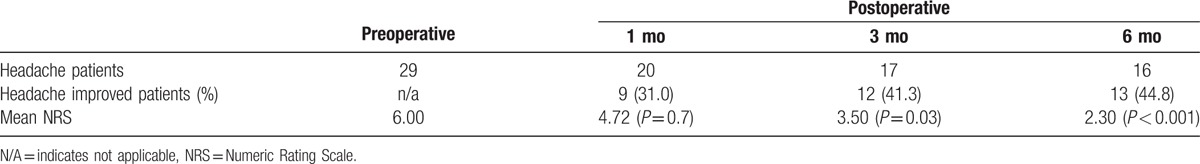
Assessment of headache at preoperative, 1 month, 3 months, and 6 months after endovascular treatment.

Three months after the procedure, 12 patients had their headache alleviation, including 7 patients reporting headache totally disappeared and 5 patients’ marked improvement. The mean headache score was 6.00 preoperatively versus 3.50 at 1 month postoperatively (*P* = 0.03).

At the time point of 6 months, 13 patients (44.8%) reported that their headache was relieved, including 6 reported a disappearance of headache, 6 answered that their headache improved markedly, and 1 patient reported a slight improvement in headache. Overall, the NRS headache scores reduced after endovascular treatment in the 29 patients with preoperative headache. The mean headache score was 6.00 preoperatively versus 2.30 at 6 months postoperatively (*P* < 0.001, Table 3).

Exploratory analyses were performed to identify potential predictors for the absence of headache improvement following aneurysm treatment (Table 4). For these analyses, patients with pretreatment headaches (n = 29) were included. The following were associated with a lack of headache improvement: having tension-type headache before treatment; having more severe headaches before treatment; stent-assisted coiling; stent implantation.

**Table 4:**
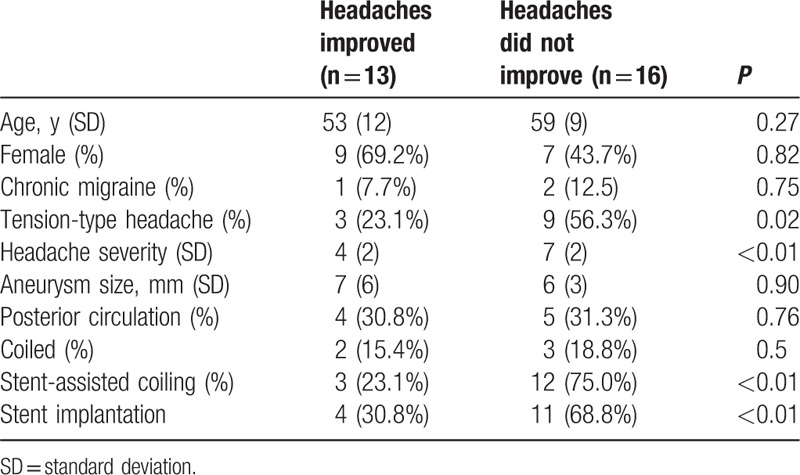
Factors associated with headache outcomes. Comparison of characteristics between patients with headache improvement following aneurysm treatment to patients with no change headaches (n = 16) revealed factors associated with headache outcomes.

## 4 Discussion

This study describes the preoperative and postoperative incidences of headache in patients with UIAs after endovascular treatment; 29 of 58 patients with UIAs (50%) presented with headache preoperatively. And there were 19 of these 29 patients, in whom recurrent headache was the complaint when he got imaging and then discovered incidental, asymptomatic UIAs. As Schwedt et al reported based on their clinical experience and available case reports, it may hint on this association was relatively authentic that patients who presenting with a thunderclap headache (often called“warning headache” or“sentinel headache”) or painful third nerve palsy often leading an asymptomatic unruptured aneurysm is identified. It is also been reported that intracranial aneurysms cause ipsilateral eye pain with radiation to the head and cause persistent headache in children^[13,14]^; intracranial internal carotid artery aneurysms cause migraine-like headache.^[12,15]^ The mechanisms that cause headache associated with an unruptured aneurysm include local thrombosis, localized meningeal inflammation, expansion of the aneurysm, dilation or stretching of intracranial arteries, and bleeding within the vascular wall.^[3,16–24]^ The intracranial arteries, especially the proximal portions of the major vessels, are innervated by trigeminal sensory nerves that could potentially be activated by aberrant blood flow and/or structural changes of the vessel wall.^[25–28]^ In general, it is generally acknowledged that headache is inseparable from the trigeminovascular theory.^[29]^

In this study, 44.8% of preoperative headaches improved 6 months after endovascular treatment, which was consistent with rate reported by previous studies. Several studies on headache outcomes after treatment of intracranial aneurysms have shown that headache severity or frequency improves in the majority of patients. Kong et al^[30]^ evaluated the effect of cerebral aneurysm treatment in patients with chronic daily headache based on the earlier ICHD-II classification and reported marked improvement in 65.3% and slight improvement in 24.5% of patients after UIA treatment. Schwedt et al^[31]^ reported decreased headache frequency in 68% of patients after aneurysm treatment, no change in 21%, and worsening headache in 11%. Choxi et al^[32]^ concluded that both surgical and endovascular treatments for UIAs are associated with a dramatic improvement in headache via a self-reported VAS at a mean of 32.4 months post-procedure. This conclusion agreed with the D-Q study, in which 76.9% of elderly patients achieved headache relief after endovascular coiling of UIAs.^[7]^ Qureshi et al^[6]^ reported Guglielmi detachable coil embolization of UIAs and a reduction of headache in 32 of 47 (68%) of patients who presented with pretreatment headache.

Patients with pretreatment tension-type headache, more severe headaches were less likely to improve following aneurysm treatment. Furthermore, stent-assisted coiling and stent implantation of the aneurysm were associated with a lack of headache improvement. Previous studies had investigated this issue, and no consensus exists regarding an association between headache improvement and associated factors. In a prospective study, Choxi et al reported that no relationship was found between the following: headache severity versus aneurysm size, sidedness of aneurysm versus sidedness of headache, and headache improvement after surgical versus endovascular treatment. Another prospective study conducted a 6 months’ follow-up, indicating that pretreatment migraine, more severe pretreatment headaches, higher pretreatment trait anxiety, and stent-assisted aneurysm coiling were associated with a lack of headache improvement. In a retrospective study, Baron et al conclude that occurrence of postprocedure headache was common after intracranial endovascular procedures and especially so with coiling and in women, smokers, and those with anxiety/depression. Hwang shows that no hypertension history and a packing attenuation of >25% are risk factors of headache development. In a retrospective study, Gu et al had demonstrated that only a preoperative headache score was associated with treatment outcome of headache, and a higher headache score predicted a lack of headache relief after endovascular treatment (*P* < 0.003). Moreover, Choi et al reported that internal carotid artery (ICA) segment aneurysm, stent-assisted coiling, and no history of hypertension were associated with post-embolization headache. In one word, it is generally acknowledged that the pretreatment headache, such as migraine or tension-type, was the important disadvantage for improvement of postprocedure headache. And the rest factors reported by authors await confirmation by more work.

But above all, we treat our limitations seriously. First, in this study, although headache was observed long-term improvement after the endovascular procedures, which is similar to those reported in the literatures. However, caution must be exercised when considering a causal relationship between headache improvement and intracranial endovascular procedures. Coil embolization may reduce the pulsatile expansion of the aneurysm sac, which leads to relief of the headache.^[6]^ There are a lot of other factors, for instance, the patient’ expectations for headache improvement following aneurysm treatment could also contribute to improvements via a placebo effect or anxiety elimination. Besides, the routine anticoagulant therapy after the intracranial treatment was 3 months’ aspirin + clopidogrel, and 3 months later, aspirin alone. That is to say, the length of aspirin’ regular administration was usually 6 months. In addition to anticoagulants, aspirin is known to be a painkiller. While, Schwedt et al reported that this daily medicine effect on headache patterns was minimal. Second, although this study placed no restrictions on the use of headache prophylactic therapy, there was no one taking headache prophylactic medicine in our patient population before procedures or during the 6-month follow-up. But it is noted that new-onset headaches occurred in 2 patients without preprocedural headache, and the headaches were severe (NRS = 7–8), they had to take nonsteroidal anti-inflammatory drugs (NSAIDs) after excluded bleeding by the head CT scan, and it was effective. Third, headache is one kind of subjective feeling and there were various sensitivities on headache in different patients; this is our inevitable limitation. Last, sample number was small in this study, but the trend has been observed in this study, which might give hint to other physicians in the future research. Further, a larger sample size, well-designed studies centered on clinical symptoms including headaches are necessary to reveal the natural history of endovascular treatment for intracranial aneurysms. Particularly, it will be more helpful to illuminate the headache changes if a well-designed study was conducted in which cerebral Digital Subtraction Angiography (DSA) group and peripheral angiography served as 2 control groups.^[33]^

## 5 Conclusion

Approximately 50% of patients had improved headache symptoms 6 months after endovascular treatment for an intracranial aneurysm compared with those pretreatment.

### 5.1 Clinical implications

1. Headache outcomes following intracranial endovascular procedures in Chinese patients with UIAs were recorded in the present study.
2. Approximately half of patients with preprocedural headache generally benefited from endovascular treatment.
3. Headache outcomes attributed to angioplasty combined with intra-arterial thrombolysis for acute occlusion of intracranial arteries should be analyzed.

## Acknowledgments

We thank all the referring clinicians and statistics professor Xinyuan Tong and the anesthetist Yajie Zhao.
